# Comparative Evaluation of the Marginal Adaptation of Cast Ni-Cr Copings, Direct Metal Laser Sintering Co-Cr Copings, and Computer-aided Design and Computer-aided Manufacturing Zr Copings: An In-vitro Study

**DOI:** 10.7759/cureus.6091

**Published:** 2019-11-07

**Authors:** Lokanathanbalaji Doddy, Sesha Reddy, Sashideepth Reddy, Narendra R

**Affiliations:** 1 Prosthodontics, Government Dental College and Hospital, Kadapa, IND

**Keywords:** marginal adaptation, scanning electron microscope

## Abstract

Background and purpose

Marginal adaptation is critical for the long-term longevity and clinical success of dental restorations. Improper marginal adaptation may lead to oral fluids, resulting in microleakage and cement dissolution. The present in vitro study aimed to evaluate the marginal adaptation of nickel-chrome (Ni-Cr) copings, cobalt-chrome (Co-Cr) coping, and zirconium (Zr) copings, produced with different manufacturing procedures.

Material and methods

A total of 45 copings were fabricated on a standardized metal die by using a two-stage putty impression and poured with die stone. They were divided into three groups of 15 each: A, B, and C. For group A, Ni-Cr copings were fabricated by conventional casting procedures; for group B, Co-Cr copings by direct metal laser sintering (DMLS); and for group C, zirconium copings by computer-aided design and computer-aided manufacturing (CAD/CAM) systems. Four areas around the tooth surface, namely, the mid-mesial, buccal, distal, and lingual surfaces, were digitally analyzed for marginal adaptation under the scanning electron microscope.

Results

The mean marginal gap for group A on the mid mesial, buccal, distal, and lingual surfaces was 79.67, 83.27, 90.67, and 89.13 µm, respectively. The mean marginal gap for group B on the mid-mesial, buccal, distal, and lingual surfaces was 38.13, 46.20, 45.73, and 42.20 µm, respectively. The mean marginal gap for group C on the mid mesial, buccal, distal, and lingual surfaces was 36.73, 31.73, 29.00, and 30.53 µm, respectively.

Conclusion

The marginal adaptation of CAD/CAM Zr copings is more accurate when compared to the DMLS Co-Cr and Cast Ni-Cr copings on a standard master die.

## Introduction

Anterior and posterior teeth have been extensively restored, with single crown and bridges, for function, speech, comfort, and aesthetics. Casting alloys have been an important part of restorative dental treatment for more than a century. Restorations commonly fabricated for fixed prosthetic treatment, such as inlays, onlays, crowns, and fixed partial dentures, are fabricated in the dental laboratory using the lost wax technique introduced by Taggart in 1907 [1-2].

Marginal adaptation is critical for long-term longevity and the clinical success of dental restorations. Improper marginal adaptation may lead to oral fluids, resulting in microleakage and cement dissolution [3-6].

The poor internal fit of a coping can increase the thickness of the cement and thus influence the mechanical stability of dental restorations. Based on a literature review, the acceptable vertical marginal gap ranges between 10 and 160 µm and the internal gap ranges between 81 and 136 µm [7-8].

The purpose of this study was to compare and evaluate the marginal adaptation of cast Ni-Cr copings, DMLS Co-Cr copings, and CAD/CAM Zr copings.

## Materials and methods

Methodology

Fabrication of a Standardized Metal Die

**Figure 1:**
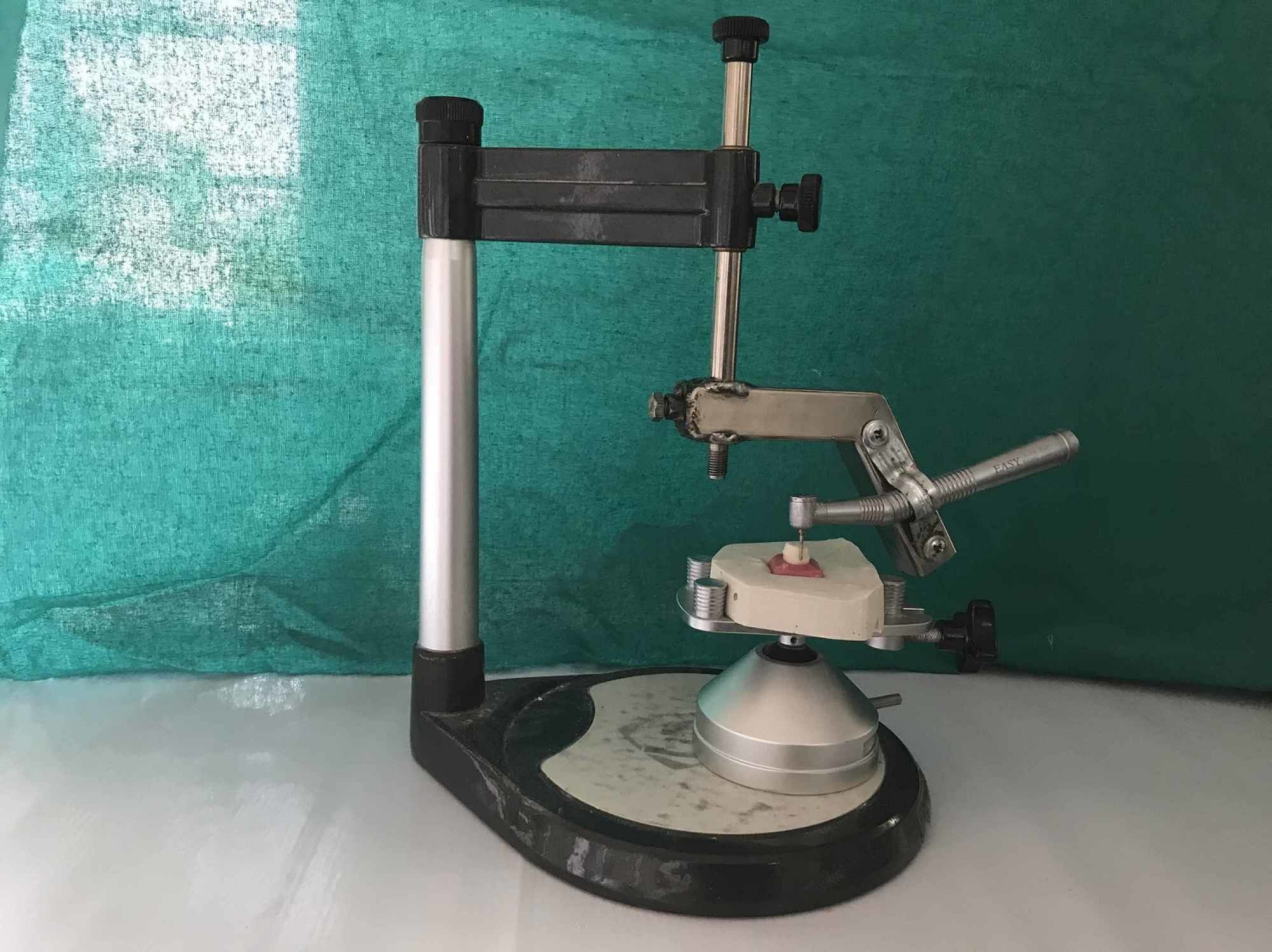
Tooth preparation on a dental surveyor

**Figure 2:**
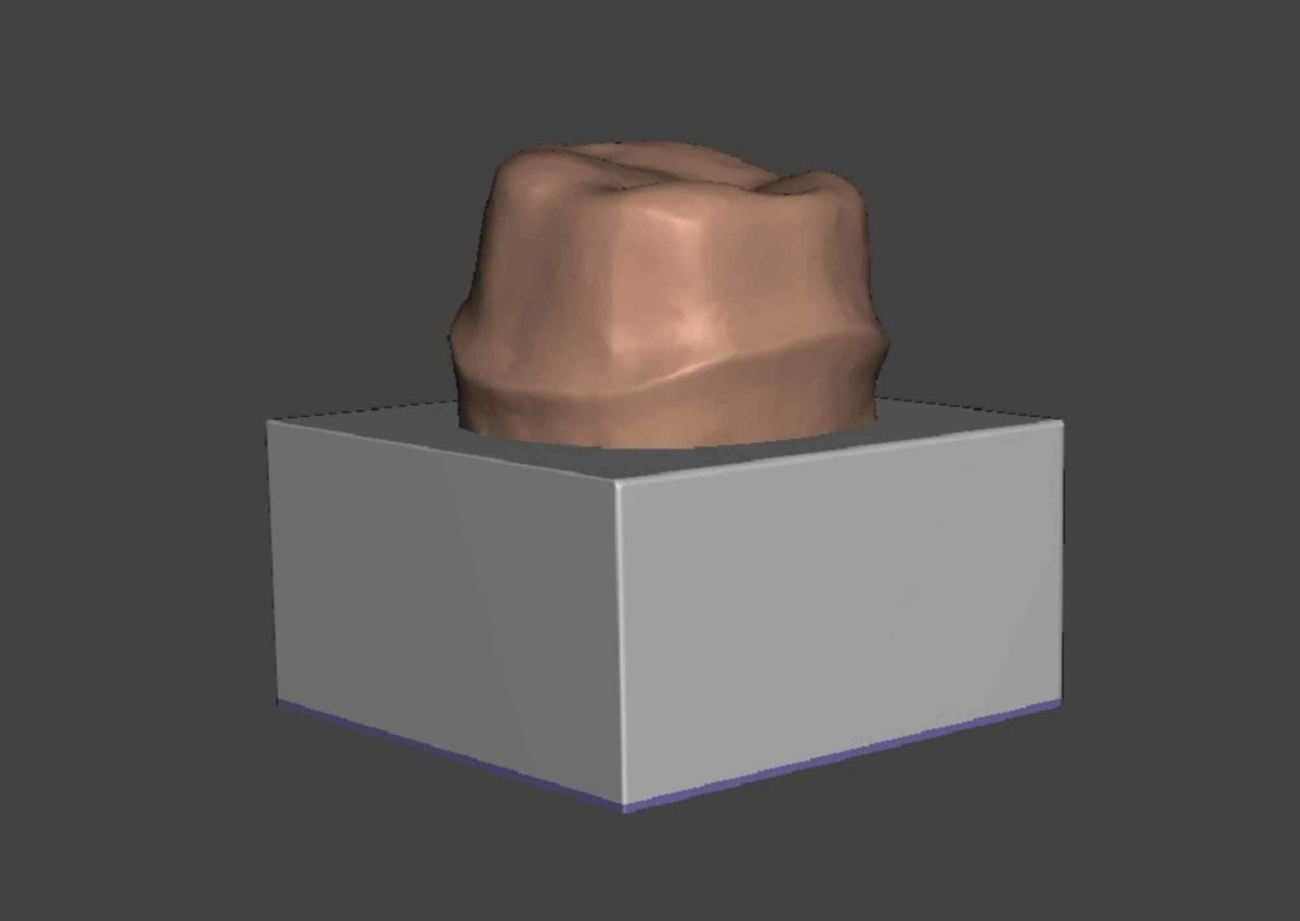
3D scanned data

In order to achieve standardized tooth preparation, a custom-made holding jig is fixed to a surveyor with a suspending arm, which was used to control the airotor orientation during tooth preparation. The movable table of the surveyor was adapted to secure the same angle of convergence for standard preparation (Figure 1). A uniform chamfer finish line of 0.5 mm in width, 6-degree occlusal convergence, 1.5 mm reduction on the functional cusp with functional cusp bevel, and 1 mm reduction on the non-functional cusp simulating a prepared mandibular first molar was scanned with a CAD/CAM machine (Sirona inEosX5; Sirona Dental Systems Inc., Long Island City, New York). The base of dimensions for the die was 15 mm x 14 mm x 5 mm (Figure 2). To fabricate a standard metal die by using CAD software data, which was then transferred to the CAD/CAN milling machine (Sirona, inLab MC X5), and obtained the metal die (Co-Cr alloy). After finishing, the polishing of the master die was carried out.

Fabrication of a Custom-made Tray and Two-stage Putty Impression

**Figure 3:**
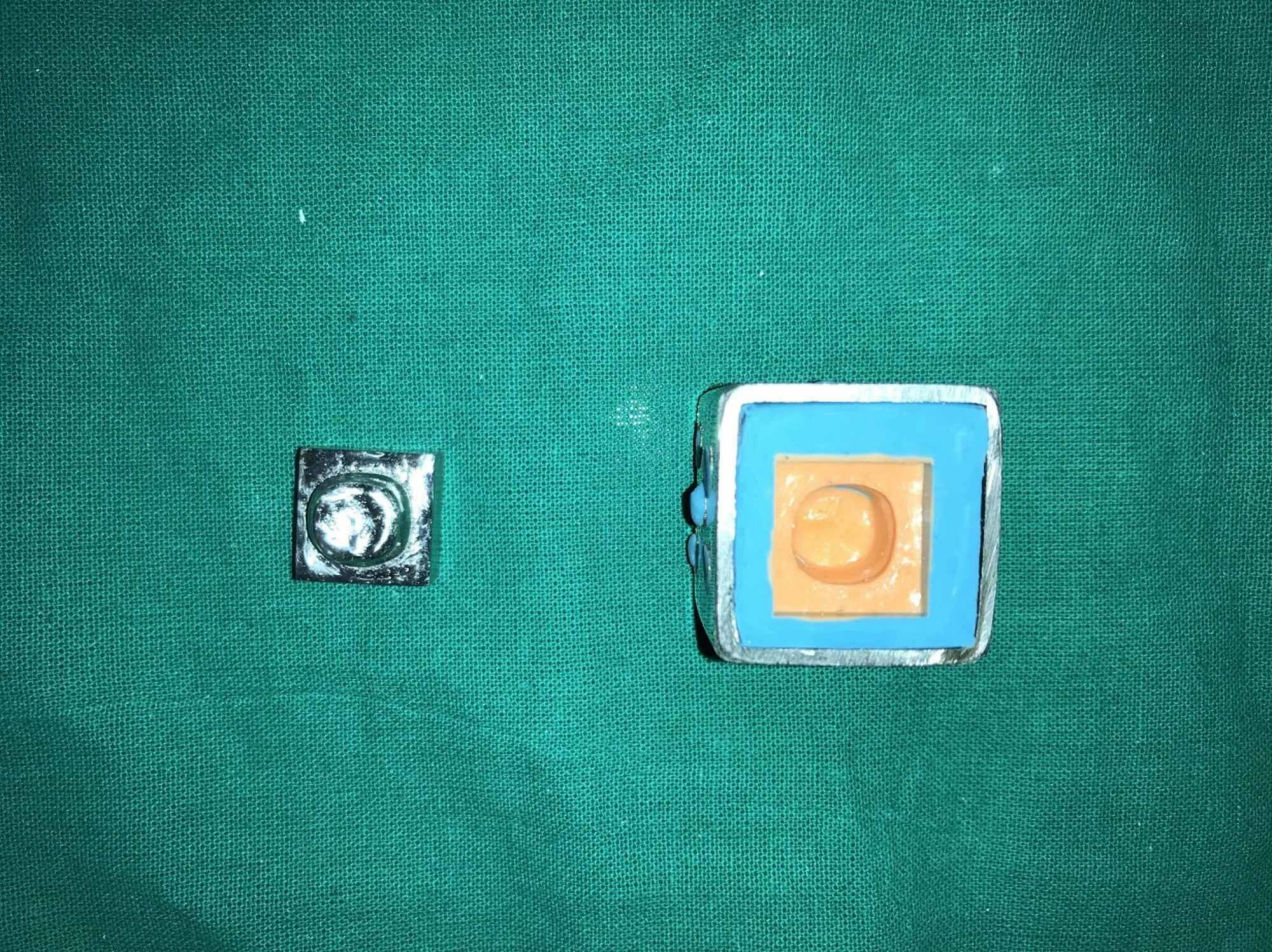
Metal die and two-stage putty impression

To standardize the impression of the metal die, a custom tray was fabricated in stainless steel (Figure 3). Holes were made on the outer surface of the square-shaped, stainless-steel custom tray for the mechanical retention of the impression material. Forty-five separate polyvinyl elastomeric siloxane impressions (Dentsply, Aquasil putty soft/light body, Germany) were made and the die stone (Type IV dental stone, Kalrock, Kalabhai Karson Pvt. Ltd., Mumbai, India) was mixed as per the manufacturer recommendation and poured into the mold using a mechanical vibrator. 

The working models were grouped as follows

1. Group A (Ni-Cr coping): Accelerated casting technique (15 samples)

2. Group B (Co-Cr coping): Direct metal laser sintering (15 samples)

3. Group C (Zirconium coping): CAD-CAM systems (15 samples)

Fabrication of Groups A, B, and C and Cementation

To fabricate the cast Ni-Cr copings, the virtual wax pattern coping thickness should be designed as 0.5 mm using the CAD software program (Sirona, inEosX5). The wax pattern was invested with a phosphate-bonded investment (Bella Bond Plus, Bego Bremer Gold Wilh. Herbst GmbH & Co. KG, Bremen, Germany) and cast with a Ni-Cr alloy (Bella Sun, Bego Bremer Gold Wilh. Herbst GmbH & Co. KG) using a centrifugal casting machine (LC cast-60, Confident Dental Equipments Pvt. Ltd., New Delhi, India).

In order to fabricate the DMLS copings, the same virtual coping design was used, as stated above, with the CAD software program. Then, the copings were fabricated using a DMLS machine (EOSINT M270, EOS GmbH, Krailling, Germany) by fusing Co-Cr powder (EOS Cobalt Chrome SP2, EOS GmbH).

**Figure 4:**
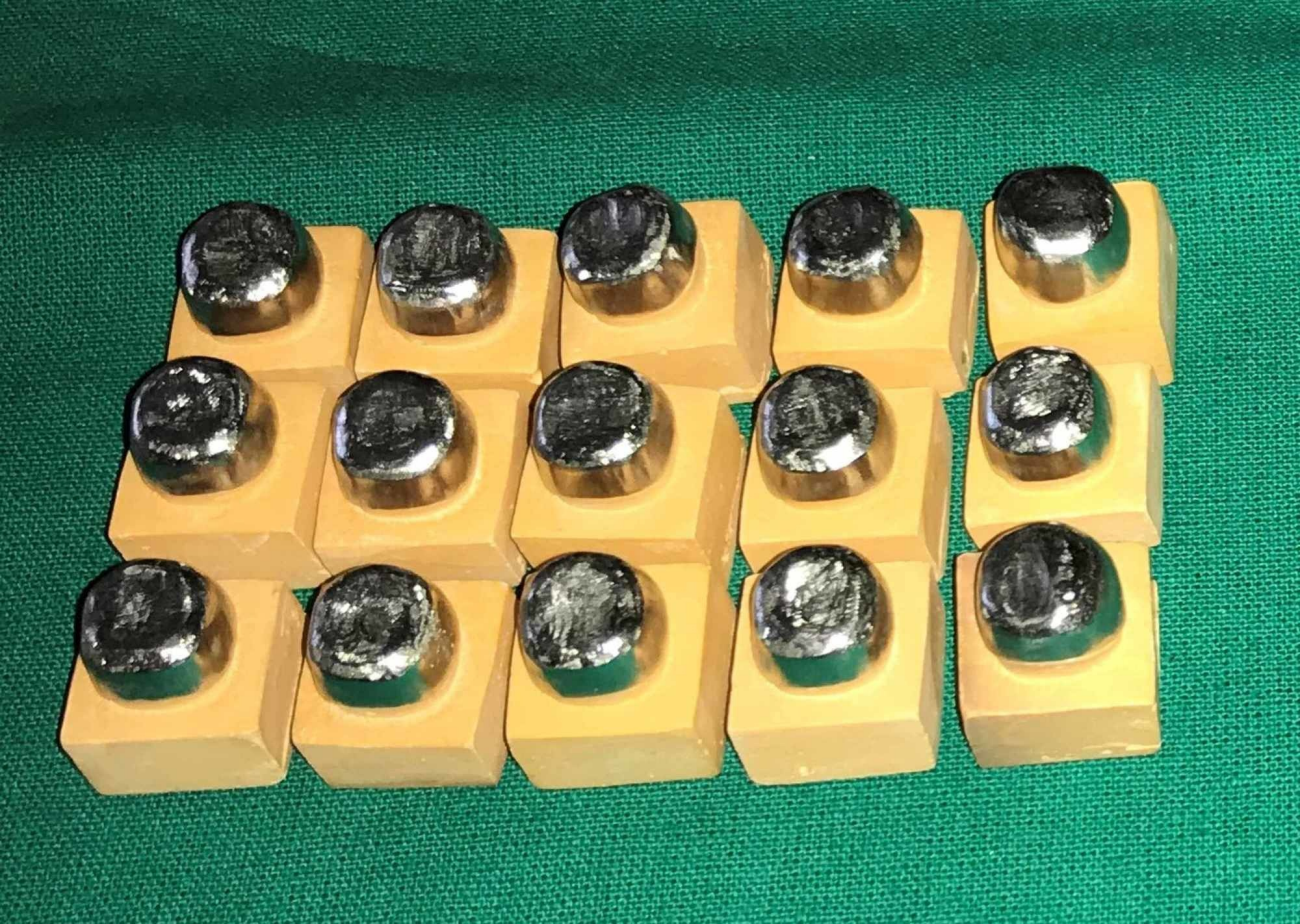
Group A Ni-Cr copings Ni-Cr: nickel-chrome

**Figure 5:**
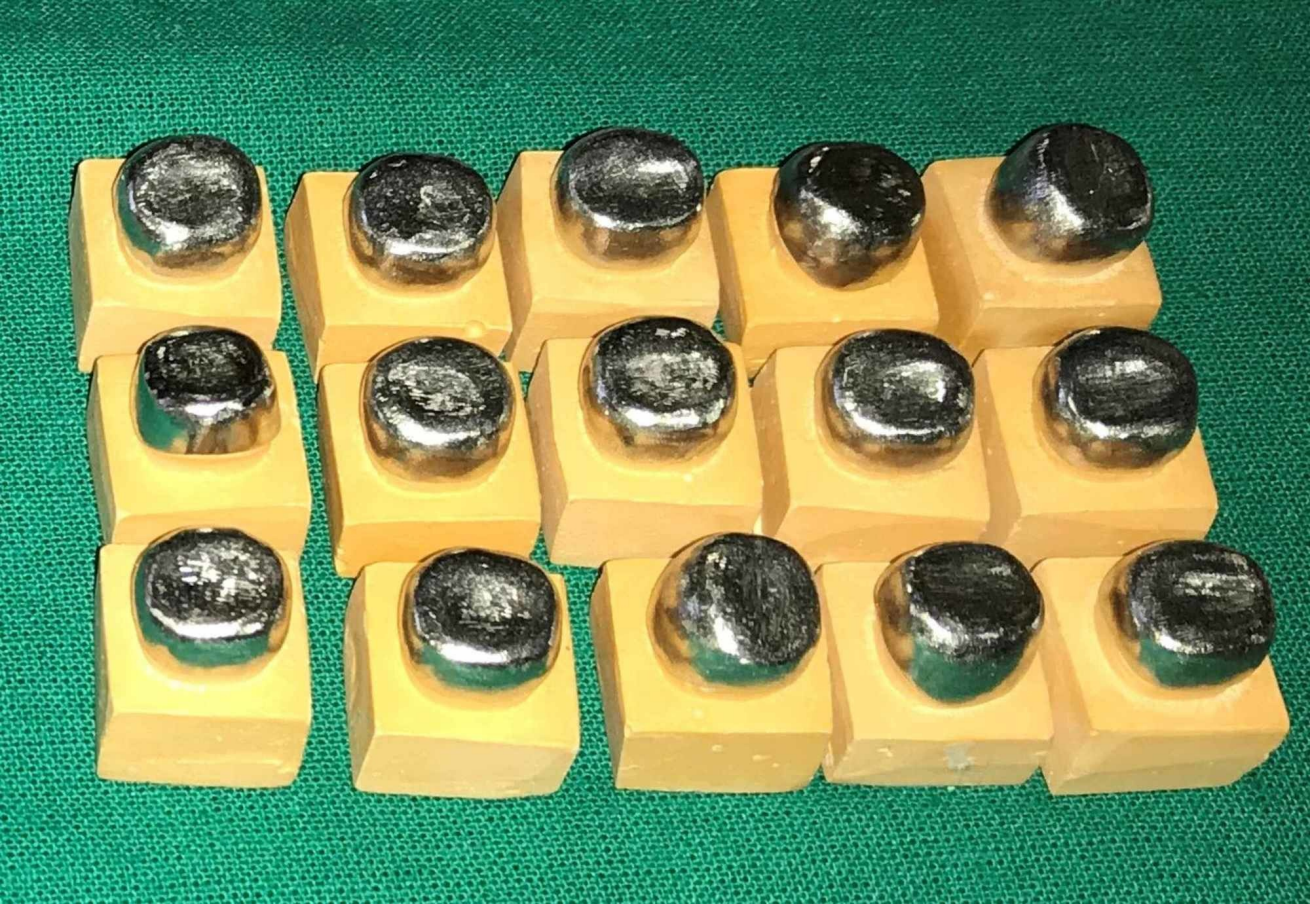
Group B Co-Cr coping Co-Cr: cobalt-chrome

**Figure 6:**
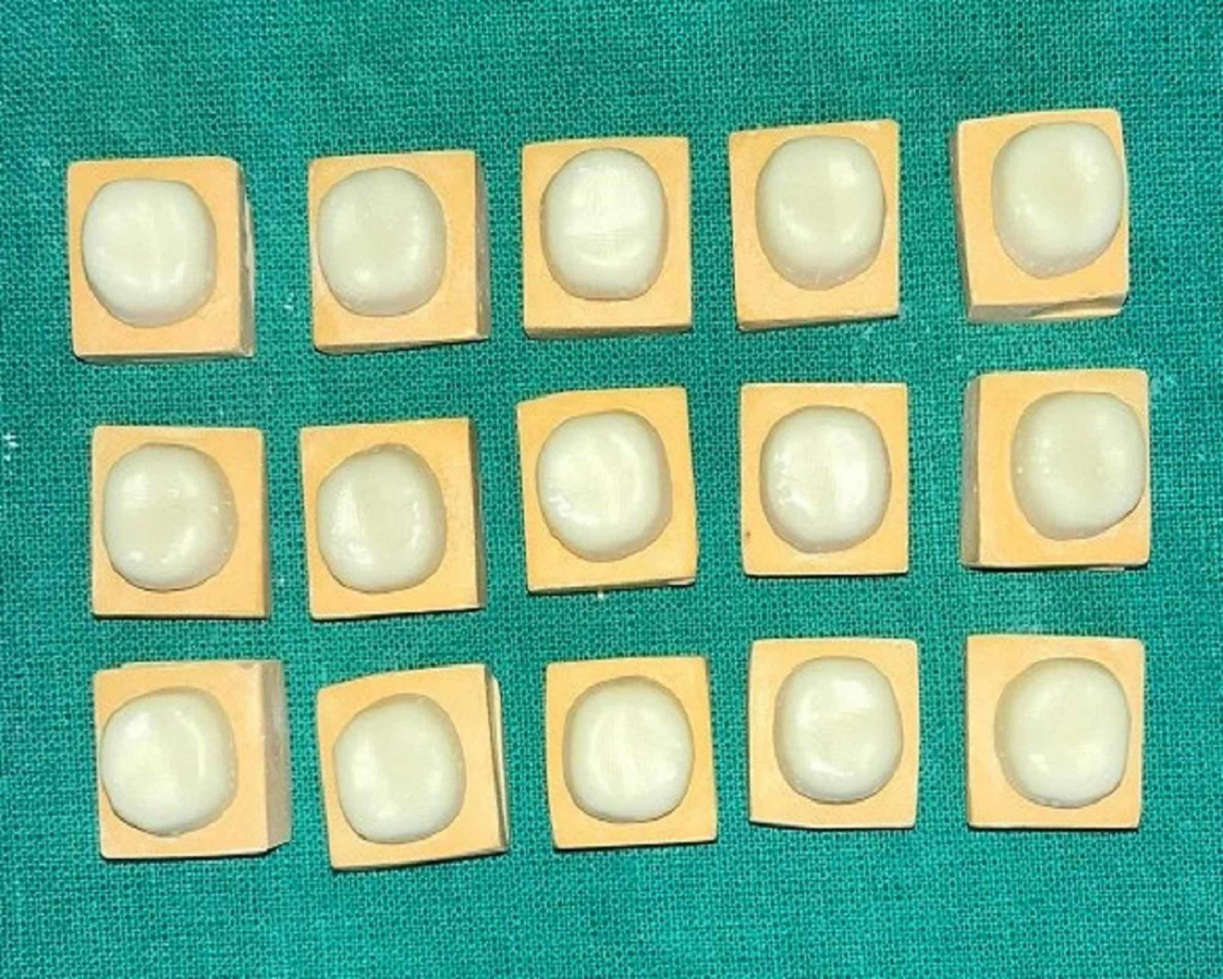
Group C Zr coping Zr: zirconium

In order to fabricate zirconium copings, the same virtual coping design was used, as stated above, with the CAD software program. Designed data were converted into processing data and sent to the processing machine (inLab MC5, Sirona). The zirconia blocks (Cercon, Yttria-stabilized zirconia) were cut and milled, and then the milled blocks were finally sintered to make zirconia coping. After finishing and polishing, groups A, B, and C copings were evaluated for marginal adaptation (Figures 4-6).

The 45 test samples were tried on the respective die stone models and inspected before the cementation procedure. A type I glass ionomer cement (GC Fuji, Tokyo, Japan) was used to lute the coping on the die (stone models), with the help of a finger until resistance was met. The excess cement was removed carefully and immediately, without damaging the margins of the die stone models.

Measurement of Vertical Marginal Adaptation Using a Scanning Electron Microscope

**Figure 7:**
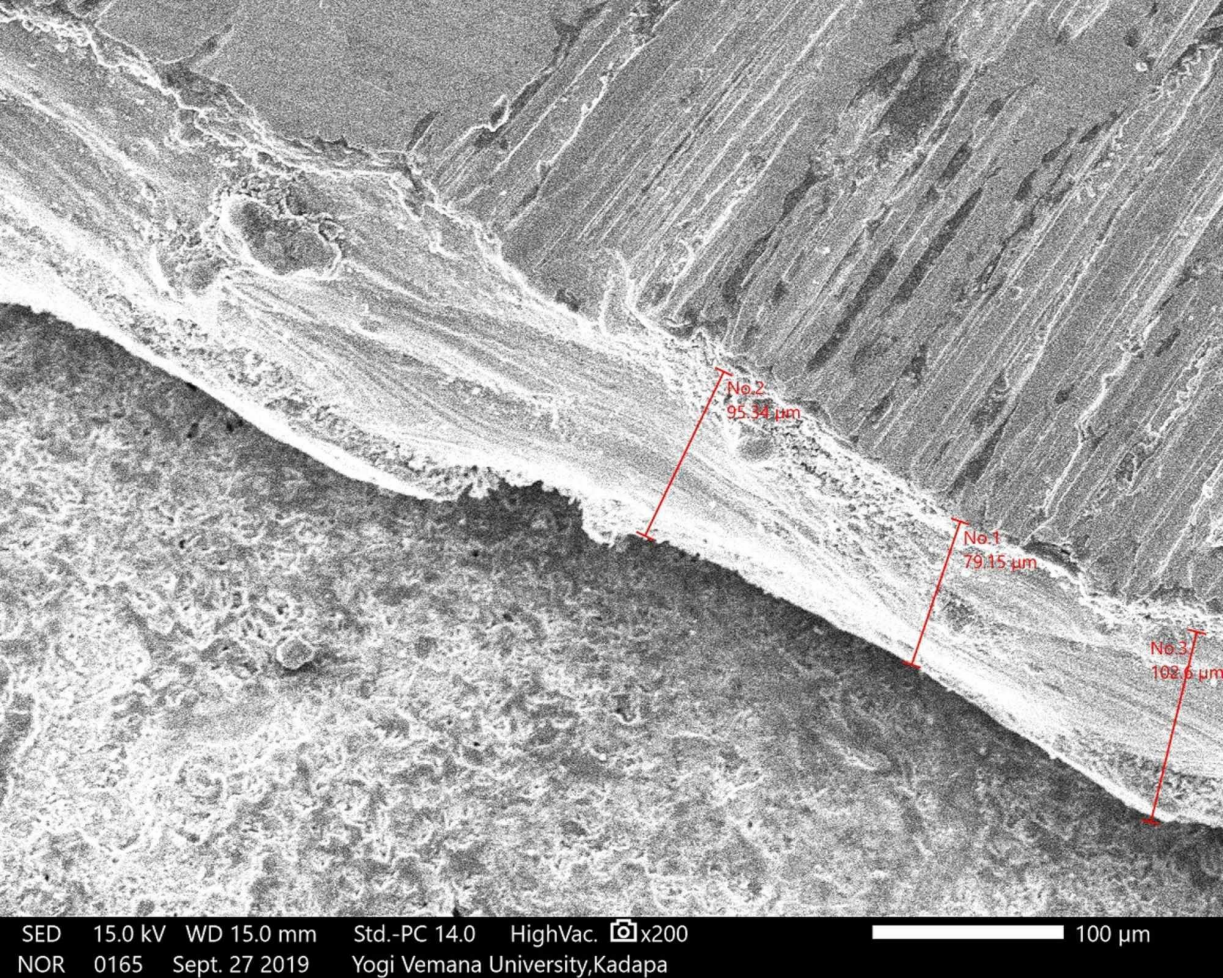
SEM analysis; group A Ni-Cr coping SEM: scanning electron microscope; Ni-Cr: nickel-chrome

**Figure 8:**
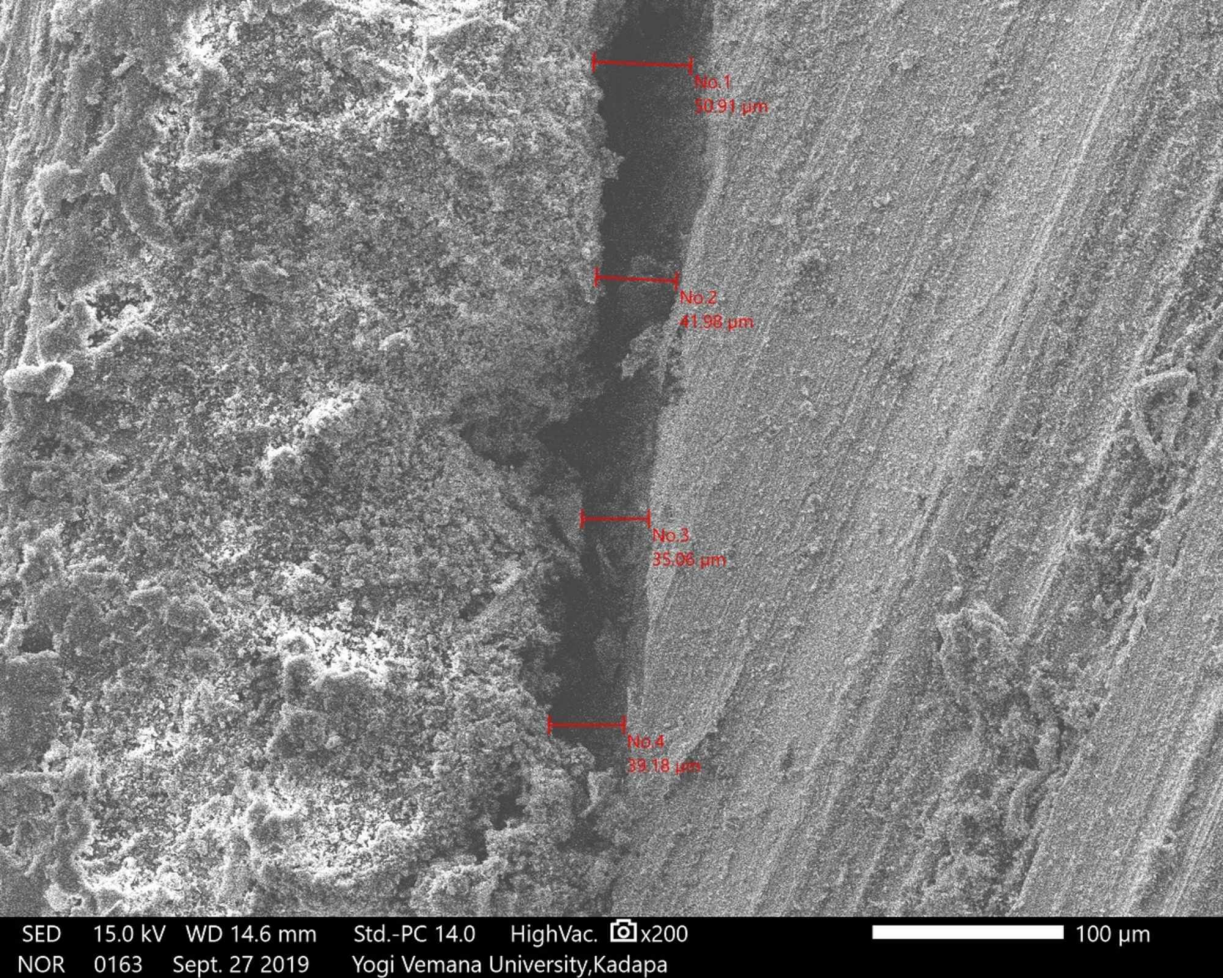
SEM analysis; group B Co-Cr coping SEM: scanning electron microscope; Co-Cr: cobalt-chrome

**Figure 9:**
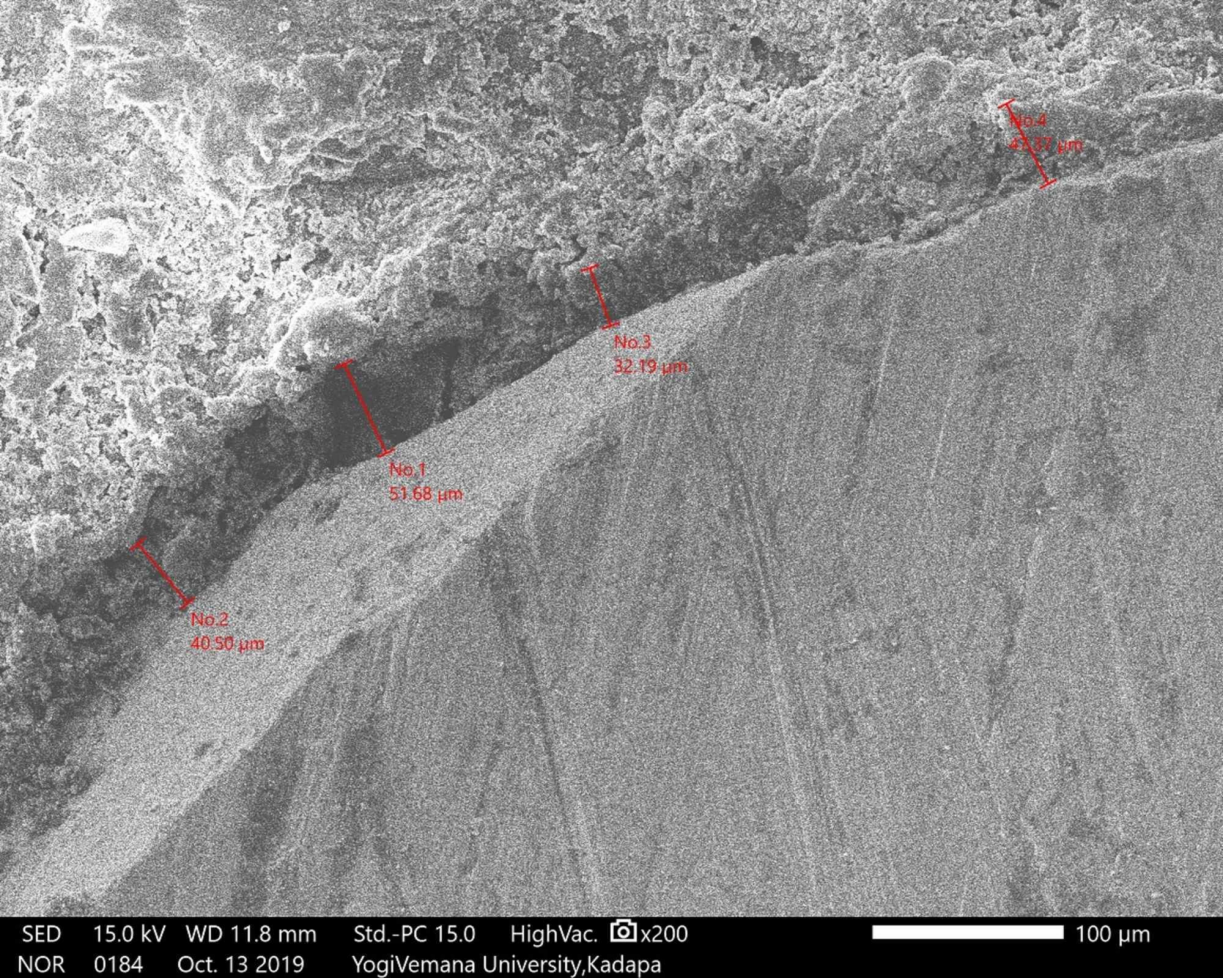
SEM analysis; group C Zr coping SEM: scanning electron microscope; Zr: zirconium

The samples were sputtered with gold for four minutes (DII-29030SCTR). After that, copings were placed in the scanning electron microscope (SEM) (JSM-IT500, JEOL, Ltd., Tokyo, Japan) at 200X magnifications using the particle measure system. Marginal adaptations were measured in four predetermined locations in the mid-mesial, mid-buccal, mid-lingual, and mid-distal surfaces of three groups of copings, respectively (Figures 7-9).

## Results

The mean ± SD marginal gap of group A on the mid-mesial, buccal, distal, and lingual surfaces was 79.67 ± 11.09, 83.27 ± 13.60, 90.67 ± 12.43, 89.13 ± 9.19 µm, respectively. The mean ± SD marginal gap of group B on the mid-mesial, buccal, distal, and lingual surfaces was 38.13 ± 6.41, 46.20 ± 8.57, 45.73 ± 7.67, 42.20 ± 5.93 µm, respectively. The mean ± SD marginal gap of group C on the mid-mesial, buccal, distal, and lingual surfaces was 36.73 ± 9.06, 31.73 ± 14.02, 29.00 ± 7.10, 30.53 ± 9.93 µm, respectively (Table 1). A comparison of the three groups (Ni-Cr copings, Co-Cr copings, and Zr copings) with the mean marginal gap in the mid-mesial, mid-buccal, mid-distal, and mid-lingual surfaces was done by one-way analysis of variance (ANOVA). The marginal adaptation of group A on the mid-mesial, buccal, distal, and lingual surfaces as compared to group B and group C was found to be statistically significant (p<0.0001). The marginal adaptation of group B on the mid-mesial, buccal, distal, and lingual surfaces as compared to group C and group A was found to be statistically significant (p<0.0001). The marginal adaptation of group C on the mid-mesial, buccal, distal, and lingual surfaces as compared to group A and group B was found to be statistically significant (p<0.0001).

**Table 1:** Comparison of three groups (Ni-Cr copings, Co-Cr copings, Zr copings) with a mean marginal gap in the mid-mesial, mid-buccal, mid-distal, and mid-lingual surfaces by one-way ANOVA Ni-Cr: nickel-chrome; Co-Cr: cobalt-chrome; Zr: zirconium; ANOVA: analysis of variance

Variables	Groups A, B, C	Min	Max	Mean	SD	SE
Mid-mesial surface	Ni-Cr copings	56.00	98.00	79.67	11.09	2.86
	Co-Cr copings	29.00	51.00	38.13	6.41	1.66
	Zirconium copings	25.00	51.00	36.73	9.06	2.34
	F-value	108.786				
	P-value	0.0001*				
Mid-buccal surfaces	Ni-Cr copings	59.00	102.00	83.27	13.60	3.51
	Co-Cr copings	29.00	58.00	46.20	8.57	2.21
	Zirconium copings	18.00	67.00	31.73	14.02	3.62
	F-value	69.8692				
	P-value	0.0001*				
Mid-distal surfaces	Ni-Cr copings	67.00	112.00	90.67	12.43	3.21
	Co-Cr copings	34.00	57.00	45.73	7.67	1.98
	Zirconium copings	17.00	42.00	29.00	7.10	1.83
	F-value	173.616				
	P-value	0.0001*				
Mid- lingual surfaces	Ni-Cr copings	67.00	102.00	89.13	9.19	2.37
	Co-Cr copings	32.00	51.00	42.20	5.93	1.53
	Zirconium copings	16.00	51.00	30.53	9.93	2.56
	F-value	198.484				
	P-value	0.0001*				

## Discussion

Marginal discrepancies in the range of 40-120 µm have been reported to be clinically acceptable with regards to the longevity of a restoration. All the copings tested in this study are in the range of 16-112 µm, which is within acceptable limits [7-11].

All the in-vitro studies were carried out with their standardized design. The method of measurement of marginal discrepancy was predominantly stereomicroscope or traveling microscope, and the optic microscope was used in their studies [11-16].

It is difficult to interpret the statistical results of previous studies because of variations in the sample size, measurements per specimens, and measurement methods used. The most common methods are direct viewing, sectioning, probing, and explorative and visual examinations. In the current study, the direct viewing of the crown on a die is used to measure the marginal fit of all the copings. Direct viewing has the advantage of being non-destructive and, therefore, applicable to clinical practice [17-19].

This in-vitro study examined the marginal adaptation of cast Ni-Cr copings, DMLS Co-Cr copings, and CAD/CAM Zr in predetermined areas, which was measured using an SEM. The marginal adaptation of CAD/CAM Zr copings is more accurate when compared to DMLS Co-Cr and Cast Ni-Cr copings on a standard master die.

Limitations

This is an in-vitro study that cannot simulate oral conditions. Finger pressure has been used for the cementation procedure of the metal copings. Though this method simulates the cementation of restorations clinically, it should be emphasized that the use of finger pressure is variable.

## Conclusions

The marginal fit of CAD/CAM zirconia copings is more accurate as compared to DMLS and cast Ni-Cr alloy copings on a standardized metal master model. The base metal alloy (Ni-Cr) exhibited a discrepancy that was significantly higher than the rest of the groups. The marginal adaptation of all the copings was within the clinically acceptable range of 80-120 µm.
